# Investigating Immunotoxicity in Black Carp (*Mylopharyngodon piceus*) Fingerlings Exposed to Niclosamide

**DOI:** 10.3390/life14050544

**Published:** 2024-04-24

**Authors:** Hao Wu, Xiping Yuan, Xing Tian, Jinwei Gao, Min Xie, Zhonggui Xie, Rui Song, Dongsheng Ou

**Affiliations:** Hunan Fisheries Science Institute, Changsha 410153, China; wh17380133463@163.com (H.W.); kerryuan@163.com (X.Y.); tianx2323@163.com (X.T.); gaojinwei163@163.com (J.G.); xieminhaha@126.com (M.X.); njdodx@163.com (D.O.)

**Keywords:** niclosamide, black carp (*Mylopharyngodon piceus*), detoxification, hepatotoxicity

## Abstract

Niclosamide (NIC) is a potent salicylanilide molluscicide/helminthicide commonly utilized for parasite and mollusc control in aquatic environments. Due to its persistent presence in water bodies, there is growing concern regarding its impact on aquatic organisms, yet this remains inadequately elucidated. Consequently, this study aims to assess the hepatotoxic effects and detoxification capacity of black carp (*Mylopharyngodon piceus*) in a semi-static system, employing various parameters for analysis. NIC was applied to juvenile black carp at three different concentrations (0, 10 and 50 μg/L) for 28 days in an environmentally realistic manner. Exposure to 50 μg/L NIC resulted in an increase in hepatic lysozyme (LYZ), alkaline phosphatase (ALP), and complement 4 (C4) levels while simultaneously causing a decrease in peroxidase (POD) activity. Additionally, NIC exposure exhibited a dose-dependent effect on elevating serum levels of LYZ, ALP, complement 3 (C3), C4, and immunoglobulin T (IgT). Notably, the mRNA levels of immune-related genes *tnfα*, *il8*, and *il6*, as well as *nramp* and *leap2*, were upregulated in fish exposed to NIC. RNA-Seq analysis identified 219 differentially expressed genes (DEGs) in *M. piceus* after NIC exposure, with 94 upregulated and 125 downregulated genes. KEGG and GO analyses showed enrichment in drug metabolism pathways and activities related to oxidoreductase, lip oprotein particles, and cholesterol transport at 50 μg/L NIC. Additionally, numerous genes associated with lipid metabolism, oxidative stress, and innate immunity were upregulated in NIC-exposed *M. piceus*. Taken together, these findings indicate that NIC has the potential to cause hepatotoxicity and immunotoxicity in *M. piceus*. This research offers important insights for further understanding the impact of molluscicide/helminthicide aquatic toxicity in ecosystems.

## 1. Introduction

Niclosamide, classified as a salicylanilide molluscicide/helminthicide, exerts its antiparasitic effects through the uncoupling of mitochondrial oxidative phosphorylation in ATP synthesis [1,2]. Due to its broad spectrum of activity, niclosamide has been extensively utilized in agricultural practices for an extended period [3]. In China, niclosamide has served as a crucial molluscicide for schistosomiasis prevention for five decades, with an annual consumption exceeding 3200 tons [4]. The accumulation of niclosamide in surface water and the surrounding environment poses a threat to ecosystems and animal health as it enters the food chain [5]. Research findings indicate that detectable levels of niclosamide residues, such as 38 μg/L in water and 474 μg/kg in sediment, have been observed near Poyang Lake [4]. This phenomenon was clearly demonstrated in a recent environmental study carried out in Dongting Lake, China, where the levels of NIC in the muscle tissue of ten commercially harvested fish varied between 20 and 2244 pg/g dw [6]. The significant presence of residues in the environment can have detrimental impacts on non-target environmental organisms, necessitating further investigation into the potentially harmful effects of niclosamide residues on aquatic organisms. Previous research has shown that niclosamide exposure can lead to impaired embryonic development, endocrine and metabolic disruptions, and oxidative damage in zebrafish [6,7,8,9]. Niclosamide has been documented to induce liver, gut, and gill toxicity in black carp [10]. Given its harmful effects on aquatic organisms, there is an anticipated increase in apprehension regarding the environmental risks associated with niclosamide. Therefore, it is imperative to develop a thorough comprehension of the mechanisms underlying niclosamide toxicity to non-target organisms.

Due to its persistence in water, niclosamide exhibits toxicity towards aquatic organisms and presents a potential risk to human health through the transfer of nutrients [11]. It is well-known that *M. piceus*, a prominent fish species in China, is particularly vulnerable to niclosamide exposure [10]. The liver, serving as a vital immunological and metabolic organ in carp, is constantly exposed to circulating antigens and endotoxins [12]. Previous research indicates that approximately 75% of niclosamide is concentrated in the liver [13]. Moreover, the liver contains a variety of immune cells, with approximately 10% of the genes identified in the fish liver transcriptome falling under the Gene Ontology (GO) category of “immune system process” [14]. Analysis of RNA-seq data in zebrafish has revealed that differentially expressed genes (DEGs) associated with the immune response are interconnected in protein interaction networks, suggesting that exposure to nicotine (NIC) may trigger immune system activation and inflammation [13]. These findings are consistent with previous research in this area [11,15].

Therefore, evaluating the effects of environmental pollutants on liver tissue serves as a viable method for assessing the safety of the aquatic environment [16,17]. Additionally, the growing concerns surrounding the harmful effects and toxic mechanisms of NIC on black carp liver highlight the need for further investigation. Analyzing alterations in gene expression following exposure to xenobiotics in a specific organism can offer valuable insights into the impacts on biochemical pathways and physiological functions in the field of ecotoxicology research [18]. High-throughput transcriptome sequencing has emerged as the predominant technique in transcriptome research and is commonly employed to investigate the deleterious effects of pharmaceutical substances on living organisms [19,20]. In this study, a chronic toxicology assessment was conducted and followed by an examination of the immunological and biochemical responses of fish exposed to environmentally relevant levels of niclosamide. Subsequently, the molecular pathways underlying these adverse effects were excavated through transcriptome analyses.

## 2. Materials and Methods

### 2.1. Ethics Statement

All of the animal procedures were carried out in strict accordance with the guidelines (license no. HNFI20221222) and approval was obtained from the animal welfare and ethics committee of Hunan Fisheries Science Institute (procedure approval 22 December 2022).

### 2.2. Niclosamide Preparation and Testing Animals

Niclosamide (NIC, Chemical Formula: C_13_H_8_Cl_2_N_2_O_4_, Cas: 50–65–7, purity ≥ 99%) was procured from Shanghai Aladdin Biochemical Technology Co., Ltd. (Shanghai, China). To prepare the stock solution of NIC, 1 g/L concentration was made by dissolving NIC in 80% hot ethanol. The resulting solution was stored in brown bottles at room temperature, ensuring protection from light, until it was diluted to the desired mass concentration for the experiment. Black carp fingerlings (body weight: 38 ± 2 g) were acquired from the breeding base of Hunan Fisheries Science Institute (Changsha, China) and kept in aerated aquariums. In the acclimation period, the fingerlings were fed twice per day with commercial fish food. The parameters for water quality were measured using a hand-held YSI meter (YSI, Yellow Springs, OH, USA) and included a photoperiod of 12 h of light followed by 12 h of darkness, a temperature of 25.00 ± 1.00 °C, a pH level of 7.4 ± 0.2, and dissolved oxygen of 6 ± 0.5 mg/L.

### 2.3. Construction of the NIC Exposure Model and Sampling

After being acclimated for two weeks, all forty-five healthy black carp (mass: 38 ± 2 g; total length: 13 ± 1 mm) were randomized into 3 groups: control, 10 μg/L NIC, and 50 μg/L NIC. To maintain the preset level of NIC concentration, the test solution was half-changed daily. High-performance liquid chromatography (HPLC) was used to determine the actual NIC levels, as provided by Lanboru Co., Ltd. (Chengdu, China). Three groups were formed and were set up in three parallel tanks, each containing 60 L of tap water and five fish fingerlings. The period of this test lasted for 28 days. The fish were fed ad libitum twice per day with commercial fish feed to apparent satiation. The black carp were fasted for 24 h after the last test. A 100 mg/L dose of MS-222 was administered to fish as an anesthetic overdose [21]. As the fish were small, the five samples for each replicate tank were merged into one Eppendorf tube after dissection, resulting in three duplicates per group. Whereafter, the blood samples were collected from the caudal vein, centrifuged (1000× *g*, 20 min, 4 °C), and stored in the supernatant at −80 °C. After blood collection, the viscera of these fish were separated. The livers were promptly removed after sacrificing the fish on ice using sterile dissecting scissors and then washed with cold, sterile saline solution. Then, the livers from five fish in each tank were combined, frozen in liquid nitrogen, and stored at −80 °C.

### 2.4. Liver and Serum Biochemical Parameters

Biochemical parameters, including lysozyme (LYZ), immunoglobulin M (IgM), immunoglobulin T (IgT), peroxidase (POD), alkaline phosphatase (AKP), complement component 3 (C3), and complement component 4 (C4) were determined using commercial kits (purchased from Jiancheng Bioengineering Institute, Nanjing, China) according to the manufacturer’s instructions. The samples were homogenized in saline and centrifuged, and the supernatant was used to measure the biochemical parameters.

### 2.5. RNA Isolation and qPCR Analysis

The total RNA was extracted from each sample using a commercial kit (RE-03014, FOREGENE, Chengdu, China), and its integrity and concentration were assessed using gel electrophoresis and a spectrophotometer (NanoDrop Technologies, Wilmington, DE, USA). The cDNA was then synthesized from the RNA using a specific kit (Cat. No. RE-03014, Foregene, Chengdu, China). Table 1 summarizes the primers and sequences of the target genes. qPCR was performed using the 2 × SYBR Green Master Mix kit following the manufacturer’s instructions. Each gene was subjected to triplicate reactions, and their gene expression levels were normalized to *β-actin*.

### 2.6. Transcriptome Analysis

The liver samples from the control group and the 50 μg/L NIC group were sent to Danyan Technology Co., Ltd. (Chengdu, China). Briefly, the total RNA from each liver sample was extracted using RNAiso Plus (Takara, Japan) following the manufacturer’s instructions. The quality and quantity of the RNA were evaluated by visualizing distinct bands corresponding to the 18S and 28S ribosomal RNA on a 1% agarose gel electrophoresis and measuring the concentration using a NanoDrop 2000 UV Spectrophotometer (Thermo Fisher, Waltham, MA, USA). Subsequently, a cDNA library was constructed and sequenced on the Illumina HiSeq platform (Shanghai Sangon Biotechnology Co., Ltd., Shanghai, China). Following sequencing, reads containing adaptor sequences, poly-N sequences, and low-quality values were filtered out. Clean reads were used for de novo transcriptome assembly with Trinity software. Transcript annotation was performed using various databases, including GO, Nr, KEGG, PFAM, and KOG. Differential gene expression (DEG) was determined based on TPM values with *padj* < 0.05 and |FoldChange| > 2. The DEGs were analyzed via bioinformatics using the KEGG pathway database (http://www.genome.jp/kegg, accessed on 9 October 2023) and GO databases (http://www.geneontology.org, accessed on 12 October 2023). A significance threshold for KEGG pathways was set at an adjusted *p*-value (*padj*) of <0.05. The reliability of the transcriptome data was confirmed through quantitative reverse-transcription PCR (qRT-PCR) analysis of 8 randomly chosen DEGs (Table S1). Each biological replicate underwent triplicate detection, and the relative expression values of the selected genes were determined using the 2^−∆∆Ct^ method, with normalization against the *β-actin* gene’s expression levels.

### 2.7. Statistical Analysis

SPSS 19.0 software (IBM, Armonk, NY, USA) was used to perform all analyses. Data are expressed as the mean ± standard error (SEM). We conducted Levene’s equal-variance test to determine whether the variance was homogeneous, as well as the Shapiro–Wilk test to determine whether the data had a normal distribution. Group means were compared using one-way analysis of variance followed by Tukey’s post hoc test. A *p* value of <0.05 was considered significant.

## 3. Results

### 3.1. Immune Biochemical Parameters of NIC Exposure in Liver and Serum

In order to examine the impact of NIC exposure on the immune system of *M. piceus*, an analysis of hepatic and serum immunological and biochemical parameters was conducted, including LYZ, POD, C3, C4, ALP, IgM, and IgT (Table 2). The results revealed that a significant increase in hepatic LYZ activities following NIC exposure, with a dose-dependent effect observed, peaking at 3056.71 U/g in fish exposed to 50 μg/L. Conversely, the POD activities exhibited a contrasting trend, showing a slight increase in the 10 μg/L NIC group but a more pronounced decrease in the 50 μg/L NIC group. Additionally, the C3 and IgM levels exposed to NIC did not show any significant variation during the entire exposure period. Furthermore, the levels of C4, ALP, and IgT in the group exposed to 10 μg/L of NIC exhibited a significant increase, whereas in the group exposed to 50 μg/L of NIC, there was a continuous but nonsignificant increase in C4 levels. The levels of ALP and IgT in the 50 μg/L NIC group returned to those of the control group.

In comparison to the control group, the serum lysozyme (LYZ) activities exhibited a gradual increase and achieved statistical significance in the 50 μg/L NIC group. Conversely, there was a noticeable trend of elevated POD activities in the serum, although none reached statistical significance. Additionally, the levels of serum C3, C4, ALP, and IgM were significantly elevated in the 50 μg/L NIC group, while no significant differences were observed in the 10 μg/L NIC group. Furthermore, similar to the findings in the liver, NIC exposure did not result in significant alterations in the serum IgM levels throughout the entire period.

### 3.2. Immune-Related Gene Expression in Liver after NIC Exposure

**Figure 1:**
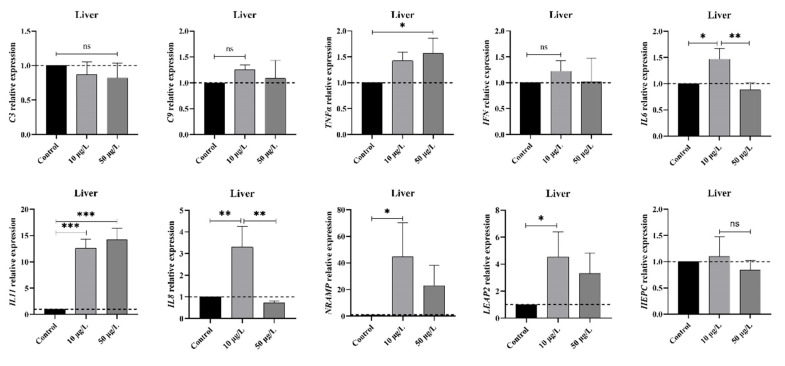
The figure shows the mRNA levels of immune markers, including *c3*, *c4*, *tnfα*, *ifn*, *il6*, *il8*, *il11*, *nramp*, *leap2*, and *hepc*, in the liver of *M. piceus* after treatment with NIC. * represent *p* < 0.05, ** represent *p* < 0.01, *** represent *p* < 0.001, ns represent not significant.

To delve deeper into the immune responses at the transcriptional level following exposure to NIC, an analysis of mRNA expression patterns of select immune-related genes was conducted utilizing the qPCR technique. The results depicted in Figure 1 reveal a notable increase in the gene expression levels of proinflammatory cytokines *tnfα*, *il8*, and *il6* within the liver of *M. piceus* in the 10 μg/L NIC group compared to the control group. Interestingly, *il8* and *il6* subsequently decreased to levels below those of the control group within the 10 μg/L NIC group. The expression levels of *il11* exhibited a dose-dependent increase, with levels 12.6- and 14.2-fold higher in the 10 and 50 μg/L NIC groups compared to the control group. Conversely, the mRNA levels of *hepc*, *c3*, *c9*, and *ifn* remained unchanged following NIC exposure across all groups. Additionally, the relative expression of *nramp* and *leap2* was significantly elevated in the 10 μg/L NIC group relative to the control group but returned to baseline levels in the 50 μg/L NIC group.

### 3.3. RNA-Seq Sequencing in Liver after NIC Exposure

**Figure 2:**
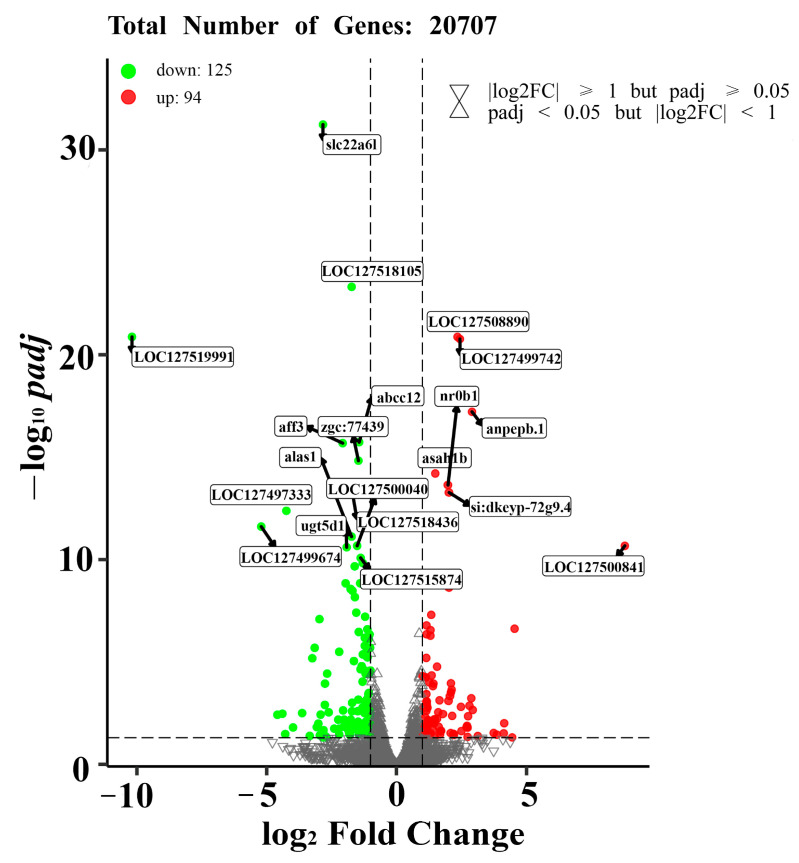
The figure shows the volcano plots of DEGs in the liver of *M. piceus* treated with50 µg/LNIC. Red and green dots indicate upregulated and downregulated genes, respectively.

**Figure 3:**
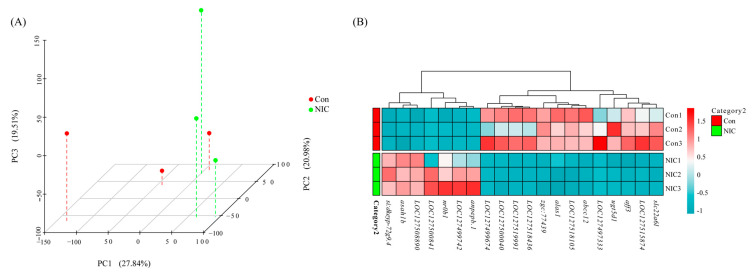
The figure shows (**A**) the principal component analysis (PCA) plotted on three dimensions for the liver gene expression profiling in two groups. PC1 explained 27.84%, PC2 explained 20.98%, and PC3 explained 19.51% of the variability; (**B**) heatmap of the top 20 highly differentially expressed genes in NIC and control samples.

Six cDNA libraries were generated and analyzed from the livers of *M. piceus* exposed to immersion in 50 μg/L NIC, as well as a control group. The characteristics of these libraries are detailed in Table S2. Following quality control procedures for the sequencing data, the number of clean reads per library ranged from 42,210,450 to 62,012,864, with a Q30 value exceeding 97.05% for each library. Subsequently, the RNA sequence data obtained in this study were deposited in the NCBI Sequence Read Archive (SRA) at http://www.ncbi.nlm.nih.gov/sra, accessed on 23 October 2023. Based on the results of differential expression analysis conducted using DESeq2 (1.16.1), a total of 219 differentially expressed genes (DEGs) were identified between the control group and the group exposed to 50 μg/L of NIC. These DEGs were visualized through the use of volcano plots, revealing that 94 genes were upregulated and 125 genes were downregulated in the 50 μg/L NIC group (Figure 2). Furthermore, principal component analysis (PCA) was performed in three dimensions to assess changes in the transcriptomes, demonstrating distinct variation between the NIC-exposed and control samples (Figure 3A). Additionally, several DEGs related to detoxification, immune response, and drug metabolism were observed in *M. piceus* following exposure to NIC (Figure 3B, Table S3).

**Figure 4:**
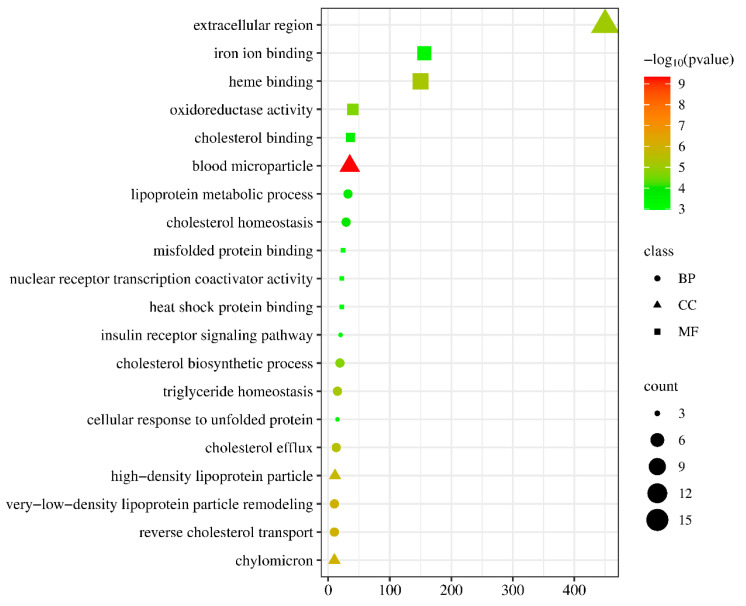
The figure shows the top 20 enriched GO terms in the liver of *M. piceus* following treatment with NIC concentration.

**Figure 5:**
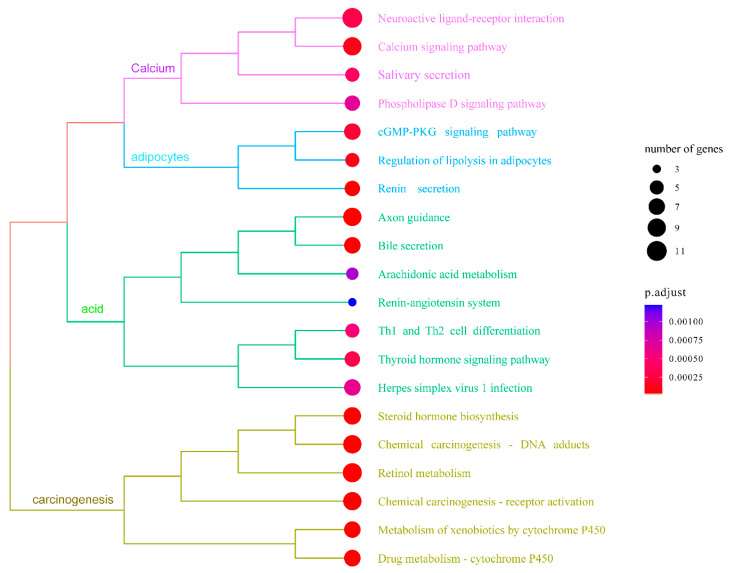
The figure shows top 20 enriched KEGG terms in the liver of *M. piceus* following treatment with NIC concentration.

In order to enhance the understanding of the biological function of DEGs, the Gene Ontology (GO) and Kyoto Encyclopedia of Genes and Genomes (KEGG) pathway enrichment analysis was conducted comparing the control and NIC groups. A total of 219 DEGs were successfully categorized into three primary groups: biological process (BP), cellular component (CC), and molecular function (MF). The most enriched GO terms in the BP category were the regulation of cholesterol biosynthetic processes (GO:0006695), followed by the regulation of cholesterol efflux (GO:0033344), cholesterol homeostasis (GO:0042632), and lipoprotein metabolic process (GO:0042157). In the CC category, the terms of extracellular region (GO:0005576), blood microparticle (GO:0072562), and high-density lipoprotein particle (GO:0034364) were also significantly enriched. In the MF category, the most enriched GO terms were heme binding (GO:0020037), iron ion binding (GO:0005506), oxidoreductase activity (GO:0016712), cholesterol binding (GO:0015485), and heat shock protein binding (GO:0031072) (Figure 4, Table S4). The KEGG pathway enrichment analysis indicated that exposure to 50 µg/L NIC resulted in the alteration of 264 pathways in *M. piceus*. Among these pathways, retinol metabolism (ko00830, 10 DEGs), drug metabolism—cytochrome P450 (ko00982, seven DEGs), metabolism of xenobiotics by cytochrome P450 (ko00980, seven DEGs), and steroid hormone biosynthesis (ko00140, eight DEGs) were significantly enriched in the 50 µg/L NIC exposure groups. (Figure 5, Table S5).

**Figure 6:**
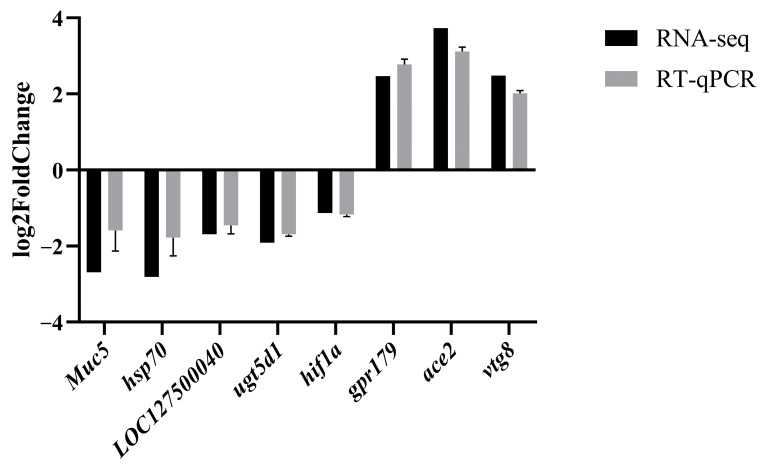
The figure shows comparison of the expression profiles of 8 selected genes as determined by RNA sequencing and qPCR.

In order to corroborate the findings of RNA-seq, a total of eight DEGs were chosen for qPCR analysis. The outcomes demonstrated a strong concordance between the gene expression levels obtained through qPCR and those obtained through RNA-seq, thereby affirming the credibility of the RNA-seq results (Figure 6).

### 3.4. Analysis of Immune and Detoxification Process in the Liver Transcriptome after NIC Exposure

In order to further elucidate the regulatory mechanisms of gene expression triggered by NIC exposure in black carp, the transcript levels of immune and detoxification-related genes are documented in Table 3. The study revealed a significant upregulation of metabolic pathways, particularly lipoprotein particle remodeling and cholesterol metabolism. The dysregulation of lipid metabolism, which can result in lipid toxicity, is closely associated with mitochondrial dysfunction and subsequent apoptosis. Notably, the apoptosis gene *bmf2* was upregulated in NIC-exposed fish, while the mRNA level of *ddit4* was downregulated. It is noteworthy that the drug metabolism–cytochrome P450 pathway, encompassing *LOC127496265* (cytochrome P450 2G1-like), *LOC127506642* (cytochrome P450 2F2-like), *LOC127510152* (cytochrome P450 family 2 subfamily K), *LOC127518105* (cytochrome P450 3A30), *LOC127500040* (cytochrome P450 1A1), *LOC127523202* (cytochrome P450 4F3), and *LOC127505748* (cytochrome b5), exhibited enrichment, with all components showing downregulation. Additionally, UDP-glycosyltransferase genes, specifically *LOC127518436* (UDP-glucuronosyltransferase 1–6) and *ugt5d1* (UDP glucuronosyltransferase 5 family, polypeptide D1), displayed a 1.9-fold decrease following exposure to NIC. Furthermore, the results revealed that the upregulation of immune-related genes, such as *LOC127518174* (tumor necrosis factor receptor superfamily member 14-like), *LOC127520149* (HERV-H LTR-associating protein 2), *LOC127500841* (MHC class I antigen), and *LOC127517132*, in the liver tissue of *M. piceus*.

## 4. Discussion

This study utilized a combination of transcriptomics analysis and assessment of physiological and biochemical traits to investigate the broader mechanisms of toxicity associated with niclosamide exposure in a non-target teleost fish species. Through an examination of the changes in biochemical parameters and gene expression profiles in the liver of *M. piceus* following 28 days of exposure, valuable insights into the molecular and biological responses of this fish to the toxicant, as well as potential detoxification pathways, were gained. As efforts to control molluscicides continue to prioritize minimizing impacts on native, non-target species, it is imperative to incorporate a thorough understanding of the potential adverse effects and pathways of elimination of molluscicides in order to develop more informed and targeted strategies for managing aquatic snails *Pomacea canaliculate* [22,23]. Consistent with prior findings, niclosamide (NIC) infiltrates the fish organism and predominantly accumulates in the liver [10,24]. The liver functions as the principal organ in fish responsible for metabolizing a variety of xenobiotics, facilitating their detoxification, and aiding in their excretion [6,25,26]. Additionally, the liver serves as one of the largest immune organs in aquatic organisms [27]. Hence, it is essential to assess the effects of niclosamide on livers in a detailed and accurate manner.

An increasing number of studies have shown that niclosamide has the potential to induce alterations in the histological architecture of liver tissue and provoke inflammation, thereby posing as risk factors for the development of liver dysfunction [11]. Exposure to niclosamide at a concentration of 50 μg/L for a duration of 28 days led to a notable increase in the expression of *tnfα*, *il8*, and *il6* genes, as well as heightened C4 and LYZ activities, indicating alterations in inflammatory and immunosuppressive responses, thereby inducing physiological stress [28]. LYZ and ALP are critical nonspecific immune enzymes involved in antimicrobial defenses and can serve as indicators of the functional status of the innate immune system [29]. The results suggest that chronic exposure to NIC led to increased hepatic and serum levels of these immune molecules in black carp, potentially resulting from metabolic dysfunction, inflammation, and innate immune activation, which are consistent with previous findings [13]. Furthermore, hepatic POD enzymes play a crucial role in the protection of teleosts by serving as antioxidants that facilitate the breakdown of hydrogen peroxide into water [27]. The findings of this study indicate a negative correlation between POD levels and niclosamide concentration, implying that niclosamide may impede POD activity. Prolonged exposure to stress can lead to an accumulation of reactive oxygen species (ROS) in tissues, leading to oxidative damage and subsequently impacting the activity of POD [30]. Research has demonstrated that minimal exposure to xenobiotics can bolster the immune response in fish, with a particular emphasis on the relationship between immune function and antimicrobial compounds [31]. The findings of this study revealed an increase in *NRAMP* and *LEAP2* mRNA levels in the liver of *M. piceus* exposed to 10 μg/L of NIC. These molecules, known as direct effectors, are crucial components of nonspecific immunity, and higher expression of antimicrobial peptide genes in other aquatic species has been linked to enhanced immune defenses [32,33]. This effect was no longer observed at higher concentrations of niclosamide. Furthermore, the components of the adaptive immune system, such as T cell receptors (TCRs), immunoglobulins (Igs), and major histocompatibility complex (MHC), may also be impacted by exposure to NIC. This analysis revealed fluctuations in hepatic and serum IgM and IgT levels, and these changes were not deemed statistically significant. Transcriptomic analyses also suggest that some DEGs involved in adaptive immune response, such as *LOC127500841* and *LOC127508899*, were upregulated in fish exposed to 50 μg/L of NIC. Taken together, these results further demonstrated that immune disorders could be induced in *M. piceus* after NIC exposure.

In aquatic organisms, there exists a complex interplay between biotransformation and the immune system, with numerous functional relationships between these two systems [34,35]. Both systems play a crucial role in enabling organisms to withstand various environmental stressors, including viruses, bacteria, and xenobiotics. This study utilized RNA-Seq analysis to examine the gene expression profiles of black carp in response to exposure to 50 μg/L of NIC. Through bioinformatics analysis, it was determined that 94 genes were upregulated and 125 genes were downregulated in liver tissues following NIC exposure. Functional analysis demonstrated that NIC exposure in *M. piceus* significantly enriched metabolic pathways such as drug metabolism-cytochrome P450 and metabolism of xenobiotics. Additionally, GO terms related to oxidoreductase activity, high-density lipoprotein particles, and cholesterol biosynthesis were prominently enriched following NIC exposure. Previous research has indicated that the detoxification of niclosamide in fish involves a variety of phase I, II, and III biotransformation pathways, which are thought to aid in the detoxification process [36,37]. The main mechanism of NIC detoxification in fishes is believed to be through phase II pathways, specifically glucuronidation mediated by UDP-glucuronosyltransferase (Ugt) [38]. Glucuronidation is the predominant method of NIC conjugation in fishes, with sulfation playing a minor role. Previous research has demonstrated the upregulation of *ugt* genes in the gills and livers of bluegill following exposure to NIC at 6, 12, or 24 h [37]. In contrast, this study indicates a significant alteration in *ugt* gene expression in response to niclosamide detoxification, specifically the downregulation of two ugt genes under niclosamide exposure. The decrease in UGT gene expression disrupts organismal homeostasis in response to hypoxic stress [39]. It is speculated that reducing the metabolism of small lipophilic molecules, such as steroid hormones, might have contributed to this phenomenon, as evidenced by the spotted scat [40]. This difference may be due to the fact that the species-specific patterns and precise mechanisms remain to be further investigated. Furthermore, research indicates that elevated levels of phase I biotransformation *cyp* gene expression during niclosamide treatment may not play a significant role in the detoxification of niclosamide [37]. The Cyp proteins functionally enhance water solubility through hydroxylation and oxidation reactions [41]. Since niclosamide already possesses a readily available hydroxyl group, the involvement of Cyp proteins in niclosamide detoxification is unlikely to be substantial, suggesting that reliance on phase II metabolism alone may be adequate for the elimination of the toxicant. Surprisingly, these findings reveal that a considerable number of *cyp* genes exhibit significant regulation following exposure to NIC in comparison to control conditions, with seven being downregulated. Analysis of the mechanistic variances in NIC detoxification between the NIC-tolerant bluegill species and the sensitive sea lamprey species indicated noticeable differences in detoxification transcriptome, particularly in genes related to phase I biotransformation, specifically those encoding *cyp* enzymes responsible for detoxifying organic compounds [42]. Hence, it is hypothesized that the *cyp* detoxification gene may have a significant impact on the detoxification of NIC in black carp. The differential expression of *cyp* genes could potentially contribute to other physiological processes responsive to niclosamide, such as energy metabolism or stress response mechanisms.

## 5. Conclusions

In summary, this study provides novel findings regarding the molecular mechanisms underlying niclosamide toxicity. Additionally, a thorough examination of gene expression levels related to alternative detoxification systems and immune responses in aquatic organisms was conducted. Exposure to environmentally relevant concentrations of niclosamide has been shown to induce significant downregulation of UDP-glucuronosyltransferase and phase I biotransformation CYP450. Subsequent research should prioritize the investigation of specific genes within immune and detoxification pathways, as well as assess the toxicological impacts of niclosamide exposure on *M. piceus* from diverse viewpoints. The broad spectrum of applications of niclosamide is anticipated to escalate its usage, exacerbating environmental contamination stemming from its use. It is crucial to prioritize the adverse impacts on wildlife and human health as a means of mitigating these effects.

## Figures and Tables

**Table 1:** The table shows the sequence of primer pairs used in the real-time quantitative PCR reaction.

Target Gene	Abbreviation	Primer Sequences (5′-3′)	Annealing Temperature (°C)
***Proinflammatory cytokines***			
*Tumor necrosis factor α*	*TNF-α*	Forward: ACGCTGCCCTTACCGAGGGT	60
		Reverse: AGGGCCACAGCCAGAAGAGC	
*Interferon α*	*IFN-α*	Forward: ATGGCTCGGCCGATACAGGA	60
		Reverse: TGGCATCCATGAGGCGGATGA	
*Interleukin 1β*	*IL-1β*	Forward: TCACTGGGAGGTGGTTCA	59
		Reverse: GAGCTGGTTTAATGGTTGTT	
*Interleukin 6*	*IL-6*	Forward: TGCCGGTCAAATCCGCATGGA	60
		Reverse: CCCGGTGTCCACCCTTCCTCT	
*Interleukin 11*	*IL-11*	Forward: CAGTACCAAAGTTGACGGATAT	60
		Reverse: CGGGAGTAGGTGGGAGTGA	
*Interleukin 8*	*IL-8*	Forward: CCTCACGGCGCGGGTTACAA	60
		Reverse: CCGCCGCAGGTTGTCAGGTG	
***Innate defense molecules***			
*Complement component 3*	*C3*	Forward: CAAGTGGCTGGTTCTCAA	56
		Reverse: ATGGCAATCACAATAAAGG	
*Complement component 9*	*C9*	Forward: TCAGAAATCACGACGACCAA	56
		Reverse: AGGGCATCCACATCATCCAA	
*Hepcidin*	*HEPC*	Forward: GCAGCCGTTCCATTCGT	56.5
		Reverse: GCCAGGGGATTTGTTTGT	
*Liver-expressed antimicrobial peptide 2*	*LEAP*	Forward: AAACCTCACGGTGCCTACT	63
		Reverse: CTCCTGCATATTCCTGTCG	
*Natural resistance-associated* *macrophage protein*	*NRAMP*	Forward: TCTGGTCTGGCGCTGTCT	63
		Reverse: AACTCTGGCTGTTCGTCGTAG	
***Housekeeping gene***			
*Beta-actin*	*β-actin*	Forward: CCAGCAGATGTGGATTAGCA	56
		Reverse: CAGTTTGAGTCGGCGTGA	

**Table 2:** The table shows alterations in LYS, POD, C3, C4, ALP, IgM, and IgT after *M. piceus* were exposed to NIC. Different lower case letters indicate significant differences between groups (*p* < 0.05).

Tissue	Parameters	Niclosamide Concentrations (μg/L)		
		Control	10 μg/L NIC	50 μg/L NIC
Liver	LYZ (U/g)	1857.51 ± 139.73 c	2587.39 ± 128.77 b	3056.71 ± 107.07 a
	POD (U/g)	7505.66 ± 136.01 a	7920.33 ± 276.36 a	6600.33 ± 330.31 b
	C3 (μg/mL)	73.03 ± 2.42 a	82.38 ± 6.09 a	83.62 ± 0.99 a
	C4 (μg/mL)	41.29 ± 1.74 b	47.21 ± 1.89 a	52.03 ± 0.99 a
	ALP (ng/g)	573.13 ± 34.96 b	747.11 ± 41.13 a	673.06 ± 28.55 ab
	IgM (ng/g)	12,538.63 ± 1500.87 a	12,040.37 ± 805.83 a	14,046.03 ± 578.79 a
	IgT (ng/g)	171.83 ± 7.91 b	201.65 ± 2.65 a	153.56 ± 7.83 b
Serum	LYZ (U/g)	858.40 ± 38.72 b	961.33 ± 33.16 ab	1073.10 ± 52.39 a
	POD (U/g)	491.67 ± 37.03 a	563. 67 ± 16.35 a	563.67 ± 35.12 a
	C3 (μg/mL)	523.78 ± 16.03 b	610.21 ± 37.82 a	668.59 ± 25.88 a
	C4 (μg/mL)	34.30 ± 0.99 b	42.47 ± 3.19 ab	46.31 ± 3.73 a
	ALP (ng/g)	582.74 ± 39.93 b	697.35 ± 60.24 ab	807.87 ± 43.82 a
	IgM (ng/g)	9579.19 ± 495.56 a	12,154.30 ± 1191.52 a	12,496.03 ± 1316.72 a
	IgT (ng/g)	142.09 ± 8.30 b	165.63 ± 9.91 ab	190.20 ± 21.66 a

**Table 3:** The table shows a summary of the DEGs involved with immune and detoxification processes in the livers of *M. piceus*.

Features	Gene Name	log2FoldChange	*p*-adj	Functions
*LOC127517132*	uncharacterized	2.083422335	<0.001	Immune response
*LOC127518174*	tumor necrosis factor receptor superfamily member 14-like	1.626307483	0.014	
*LOC127520149*	HERV-H LTR-associating protein 2	2.799184351	0.001	
*LOC127500841*	MHC class I antigen	8.785602796	<0.001	
*LOC127508899*	MHC class II antigen	−2.729012249	0.042	
*LOC127500693*	uncharacterized	−2.966246836	<0.001	
*LOC127496265*	cytochrome P450 2G1-like	−1.208747206	<0.001	Detoxification
*LOC127506642*	cytochrome P450 2F2-like	−1.160051571	0.002	
*LOC127510152*	cytochrome P450 family 2 subfamily K	−1.959354958	<0.001	
*LOC127518105*	cytochrome P450 3A30	−1.727033364	<0.001	
*LOC127500040*	cytochrome P450 1A1	−1.698724375	<0.001	
*LOC127523202*	cytochrome P450 4F3	−1.222647819	<0.001	
*LOC127505748*	cytochrome b5	−1.014338096	<0.001	
*LOC127518436*	UDP-glucuronosyltransferase 1–6	−1.515533776	<0.001	
*ugt5d1*	UDP glucuronosyltransferase 5 family, polypeptide D1	−1.917067215	<0.001	
*slc35g1*	solute carrier family 35 member G1	−1.071229676	<0.001	
*slc35f2*	solute carrier family 35, member F1/2	2.707230679	0.013	
*abcc12*	ATP-binding cassette, sub-family C (CFTR/MRP), member 12	−1.436705268	<0.001	
*abcc2*	ATP-binding cassette, sub-family C (CFTR/MRP), member 2	−1.036890355	<0.001	
*LOC127509173*	hemoglobin cathodic subunit alpha-like	1.608493192	0.003	
*bmf2*	BCL2 modifying factor 2	1.657943074	<0.001	Apoptotic process
*ddit4*	DNA-damage-inducible transcript 4	−2.753519746	<0.001	

## Data Availability

Research data are not shared.

## Supplementary Materials

The following supporting information can be downloaded at: https://www.mdpi.com/article/10.3390/life14050544/s1. Table S1: The PCR primers employed for validation of the accuracy of RNA-seq; Table S2: The quality control summary of RNA-seq clean data from *M. piceus* livers; Table S3: The top 20 highly differentially expressed genes in the liver of *M. piceus* following treatment with NIC concentration; Table S4: The top 20 enriched GO terms in the liver of *M. piceus* following treatment with NIC concentration; Table S5: The top 20 enriched KEGG terms in the liver of *M. piceus* following treatment with NIC concentration.
