# Supplementary material for: Investigating Immunotoxicity in Black Carp (Mylopharyngodon piceus) Fingerlings Exposed to Niclosamide
Source: Life (Basel). 2024 Apr 24;14(5):544. doi: 10.3390/life14050544 (PMC11122308; doi:10.3390/life14050544)
Supplement: Supplementary file 1 [file life-14-00544-s001.zip › life-2940541-supplementary.pdf]

Table S1 The PCR primers employed for validation of the accuracy of RNA-seq.

Gene	Nucleotide sequence (5′ - 3′)
Muc5	Forward: TGTGTGAGCATGGGGTGTATA; Reverse: CTGTTGAACTTGCTCTCCAGG
hsp70	Forward: GTTCCAGCCTATTTCAATGAC; Reverse: ATGTCCTTCTTGTGCTTCCTC
LOC127500040	Forward: CAAGGACAACATCCGAGA; Reverse: TAGACGACACCCCAAGAC
ugt5d1	Forward: GGATGGAAGCCATTGGGTCA; Reverse: CAAGTCCAGAACCTTCGCCT
hif1a	Forward: GACTGTAGCAGACCCTGTCCTC; Reverse: AGAGAGGGGTGAGAGAGGTAGC
gpr179	Forward: CAAGTCCAGAACCTTCGCCT; Reverse: GACTGAGTAACCACCCTGGC
ace2	Forward: CATGGAGTGGTTGAAGGAGG; Reverse: ATGTCATTTGCGTTCCAGGTG
vtg8	Forward: ; Reverse:

Table S2 The quality control summary of RNA-seq clean data from *M. piceus* livers.

Sample	total reads	total bases	q20 bases	q30 bases	q20 rate	q30 rate	GC content
Con1	53194938	7333719284	7269716354	7121343050	0.991273	0.971041	0.441067
Con2	50507236	7038299697	6976223657	6831889308	0.991180	0.970673	0.442533
Con3	49170784	6742788825	6684234752	6548107089	0.991316	0.971127	0.436940
NIC1	42210450	5901843400	5849677540	5727767509	0.991161	0.970505	0.445428
NIC2	53486444	7380308561	7316129175	7165805680	0.991304	0.970936	0.441350
NIC3	62012864	8598992874	8523210304	8347367774	0.991187	0.970738	0.445301

Table S3 The top 20 highly differentially expressed genes in the liver of *M. piceus* following treatment with NIC concentration.

**Features**	**log2FoldChange**	**Genes name**
Slc22a6l	-2.8314148	solute carrier family 22 member 6, like
LOC127515874	-1.379350875	/
Aff3	-2.072885628	AF4/FMR2 family, member 3
Ugt5d1	-1.917067215	UDP glucuronosyltransferase 5 family, polypeptide D1

LOC127497333	-4.242419811	nuclear receptor coactivator 7-like
Abcc12	-1.436705268	ATP-binding cassette, sub-family C (CFTR/MRP), member 12
LOC127518105	-1.727033364	cytochrome P450 3A30
Alas1	-1.729993848	aminolevulinate, delta-, synthase 1
Zgc:77439	-1.465234396	/
LOC127518436	-1.515533776	UDP-glucuronosyltransferase 1-6
LOC127519991	-10.19016147	/
LOC127500040	-1.698724375	cytochrome P450 1A1
LOC127499674	-5.20996132	NAD(P) transhydrogenase, mitochondrial-like
Anpepb.1	2.89701828	alanyl (membrane) aminopeptidase b, tandem duplicate 1
LOC127499742	2.431376745	ADP-ribosylation factor 4
Nr0b1	1.973350957	nuclear receptor subfamily 0, group B, member 1
LOC127500841	8.785602796	class I histocompatibility antigen, F10 alpha chain
LOC127508890	2.339228177	galectin-9-like
Asah1b	1.495228526	N-acylsphingosine amidohydrolase (acid ceramidase) 1b
Si:dkeyp-72g9.4	2.014206852	/

Table S4 The top 20 enriched GO terms in the liver of *M. piceus* following treatment with NIC concentration.

**GO ID**	***p.adjust***	**Description**	**Count**	**Category**
GO:0072562	4.76E-10	Blood microparticle	8	Cellular component
GO:0005576	8.14E-06	Extracellular region	15	Cellular component
GO:0042627	1.05E-06	Chylomicron	4	Cellular component
GO:0034364	1.63E-06	High-density lipoprotein particle	4	Cellular component
GO:0070328	6.57E-06	Triglyceride homeostasis	4	Biological Process
GO:0006695	1.82E-05	Cholesterol biosynthetic process	4	Biological Process
GO:0034372	1.05E-06	Very-low-density lipoprotein particle remodeling	4	Biological Process

GO:0042632	0.000104	Cholesterol homeostasis	4	Biological Process
GO:0042157	0.000154	Lipoprotein metabolic process	4	Biological Process
GO:0043691	1.05E-06	Reverse cholesterol transport	4	Biological Process
GO:0034620	0.00026	Cellular response to unfolded protein	3	Biological Process
GO:0033344	3.49E-06	Cholesterol efflux	4	Biological Process
GO:0008286	0.00063	Insulin receptor signaling pathway	3	Biological Process
GO:0030374	0.000841	Nuclear receptor transcription coactivator activity	3	Molecular function
GO:0031072	0.000841	Heat shock protein binding	3	Molecular function
GO:0051787	0.001091	Misfolded protein binding	3	Molecular function
GO:0005506	0.000399	Iron ion binding	7	Molecular function
GO:0015485	0.000246	Cholesterol binding	4	Molecular function
GO:0016712	2.24E-05	Oxidoreductase activity	5	MF
GO:0020037	5.95E-06	Heme binding	9	MF

Table S5 The top 20 enriched KEGG terms in the liver of *M. piceus* following treatment with NIC concentration.

**KEGG ID**	***p.adjust***	**Description**	**Count**
ko05204	5.43E-12	Chemical carcinogenesis - DNA adducts	9
ko00830	4.32E-11	Retinol metabolism	10
ko00982	3.10E-09	Drug metabolism - cytochrome P450	7
ko00140	1.33E-08	Steroid hormone biosynthesis	8
ko00980	3.22E-08	Metabolism of xenobiotics by cytochrome P450	7
ko04976	2.35E-06	Bile secretion	7
ko04360	4.57E-06	Axon guidance	9
ko05207	4.87E-06	Chemical carcinogenesis - receptor activation	9
ko04924	8.89E-06	Renin secretion	6
ko04923	8.38E-05	Regulation of lipolysis in adipocytes	5

ko04020	8.60E-05	Calcium signaling pathway	9
ko04022	0.000187	cGMP-PKG signaling pathway	7
ko04919	0.000269	Thyroid hormone signaling pathway	6
ko04080	0.000303	Neuroactive ligand-receptor interaction	11
ko04970	0.000405	Salivary secretion	5
ko04658	0.000501	Th1 and Th2 cell differentiation	5
ko05168	0.000635	Herpes simplex virus 1 infection	7
ko04072	0.000673	Phospholipase D signaling pathway	6
ko00590	0.000968	Arachidonic acid metabolism	4
ko04614	0.001242	Renin-angiotensin system	3
